# Brain metastases admissions in Sweden between 1987 and 2006

**DOI:** 10.1038/sj.bjc.6605373

**Published:** 2009-10-13

**Authors:** K E Smedby, L Brandt, M L Bäcklund, P Blomqvist

**Affiliations:** 1 Clinical Epidemiology Unit, Department of Medicine Solna, Karolinska Institutet Solna, Stockholm SE-171 76, Sweden; 2 Department of Oncology, Karolinska University Hospital Solna, Stockholm SE-171 76, Sweden; 3 Department of Oncology-Pathology, Karolinska Institutet, Karolinska University Hospital Solna, Stockholm SE-171 76, Sweden

**Keywords:** brain metastases, lung cancer, breast cancer, survival

## Abstract

**Background:**

Brain metastases (BM) constitute the most common intracranial tumours and are associated with considerable morbidity and mortality. Population-based studies of the epidemiology and time trends of BM are scarce.

**Methods:**

A population-based cohort of patients admitted to hospital with BM in Sweden between 1987 and 2006 (*n*=15 517) was identified and linked to nationwide registers of cancer incidence and death. Primary cancer types were assessed and time to hospitalisation and death was computed.

**Results:**

The annual age-adjusted incidence rate of hospitalisation for BM doubled from 7 to 14 patients per 100 000 between 1987 and 2006. The most common primary tumours among women were lung (33%), breast (33%) and colorectal cancer (7%), and among men lung cancer (44%), malignant melanoma (12%) and colorectal cancer (9%). The increase was most evident for BM patients with lung cancer (both sexes) and breast cancer (women). Survival was short, with a median of 2.7 months. It varied little by cancer type and did not improve over calendar time.

**Conclusion:**

The number of patients admitted with BM has increased rapidly in Sweden. In spite of recent improvements in the prognosis of common primary cancer types, any parallel improvement among patients with advanced cancer and BM is not indicated.

Brain metastases (BM) constitute the most common type of intracranial neoplasms (Gavrilovic and Posner, 2005), and in the US alone, more than 100 000 persons are diagnosed annually (Nathoo *et al*, 2004). The incidence of BM is believed to be increasing, not only because of the advances in the treatment of patients diagnosed with primary malignancies and decreased mortality but also because of the technical advances in neuroimaging (Barnholtz-Sloan *et al*, 2004; Gavrilovic and Posner, 2005). Although the evidence of such a trend is limited (Schouten *et al*, 2002), a future increase may be expected because of an increasingly large pool of prevalent cancer patients at risk of developing metastatic disease.

The cancer types most frequently recurring with BM are lung, breast, kidney and colorectal carcinomas, and malignant melanomas (Schouten *et al*, 2002; Barnholtz-Sloan *et al*, 2004). Patients with lung cancer appear to have the highest propensity for BM (Hochstenbag *et al*, 2000; Schouten *et al*, 2002). However, studies of the incidence and/or survival of patients with BM are often confined to one single primary cancer type. They constitute single-centre experiences or are based on autopsied patients (Johnson and Young, 1996; Nussbaum *et al*, 1996; Schouten *et al*, 2002), making incidence estimates uncertain and comparisons between tumour types difficult. Population-based investigations are either old (Guomundsson, 1970; Walker *et al*, 1985) or confined to major cancer types (Barnholtz-Sloan *et al*, 2004). Consequently, there is a need for better understanding of the contemporary epidemiology of patients with BM and recent time trends in population-based settings.

We used Swedish national population-based high-quality health-care registers to investigate patient characteristics, types of primary malignancies, survival and time trends in a cohort of 15 517 patients hospitalised with BM during a 20-year period.

## Materials and methods

### Patients

We identified a cohort of individuals with BM in the Swedish National Patient Registry (National Patient Registry, 2009). This register holds individualised information on inpatient care county-wise since 1964 and nation-wide since 1987. For every hospital discharge, dates of admission and discharge, and up to eight discharge diagnoses are recorded according to the ICD classification, and the total number of register dropouts for somatic care has been estimated to be <2%. As there is practically no private inpatient care in Sweden, the registration of hospital admissions is population-based and independent of private health insurances or socioeconomic status. The cohort consisted of all patients hospitalised with BM as a primary or secondary diagnosis in Sweden from 1 January 1987 to 31 December 2006. The ICD codes used were C793 (ICD10) and 198D (ICD9), representing metastases originating in the brain or meninges. The date of the first hospital admission was used as the index date for each patient.

To obtain information on the primary cancer site, the patients in the cohort were matched to the National Cancer Registry (NCR) (National Cancer Registry, 2009) by the national registration numbers assigned to all Swedish citizens. The NCR was established in 1958 and is estimated to be >95% complete overall, with some variation by primary cancer type (Barlow *et al*, 2009). We initially identified 16 175 patients admitted with BM in the NPR. However, following matching with the NCR, we excluded 445 patients who were concomitantly registered with a primary brain tumour. We also excluded 213 individuals with a record of a haematopoietic malignancy, as their admission for BM may represent malignant central nervous system (CNS) involvement that is clinically and biologically distinct from metastases of solid cancers. Hence, 15 517 individuals with BM were included in the final study cohort. Of these, 12 307 patients (79%) were recorded in the NCR with one primary solid cancer diagnosis either preceding the index date of admission to hospital for BM (no maximum time limit was applied), or during the first 180 days following this date. An additional 1424 individuals (9%) had records of more than one primary cancer type. For these patients, we chose the cancer type diagnosed closest in time to the index date as the most probable primary cancer. For 1786 individuals (12%), there was no record of a primary solid cancer in the NCR during the stipulated time interval around BM admission. Among these, 13 individuals were however recorded in the NCR with a first solid cancer more than 1 year after first hospitalisation for BM, 184 with benign tumours only, 492 with BM only, and 1097 were not found. In spite of the lack of NCR records of a primary malignancy for these 1786 individuals, almost half had a concomitant admission diagnosis of a primary cancer listed in the NPR. However, as the specificity of the primary cancer type in the NPR may be less valid, these were left aside.

Finally, the cohort of BM patients was matched through their national registration numbers to the Cause-of-Death Registry. This national register holds computerised information on dates and causes of death for Swedish residents since 1952 and the completeness exceeds 99% (National Cause-of-Death Registry, 2009). All individuals were followed from the index date of first admission with BM to the date of death, or 31 December 2006, whichever came first. The national registers used are maintained by the Centre of Epidemiology at the Swedish National Board of Health and Welfare. The study was approved by the Stockholm Regional Research Ethics Board (Dnr 2007/1112-31/2).

### Statistical analyses

The incidence rate of patients admitted with BM per 100 000 Swedish residents and year during 1987 to 2006 was standardized to the age distribution of the population in 1995. The time from the primary solid cancer diagnosis to the index date of first admission, as well as the time from the index date to death, was computed as the median time in months with interquartile range, and was stratified by sex, age, type of primary cancer and calendar period. The assessment of time from the primary solid cancer diagnosis to the index date was restricted to the group of patients with only one recorded primary cancer. In this group, we also used a multivariable Cox proportional hazards model to compute hazard ratios (HRs) and 95% confidence intervals (CIs) as a complementary analysis for comparison of the time between the primary cancer diagnosis and BM admission by cancer type and patient characteristics. The multivariate model included age, sex and calendar period. Survival was analysed as the median survival time in months, as 1-year survival proportions using the Kaplan–Meier method, and in a multivariable Cox proportional hazards model to estimate the relative risk (HR and 95% CI) of death according to primary cancer types and other covariates.

## Results

**Figure 1:**
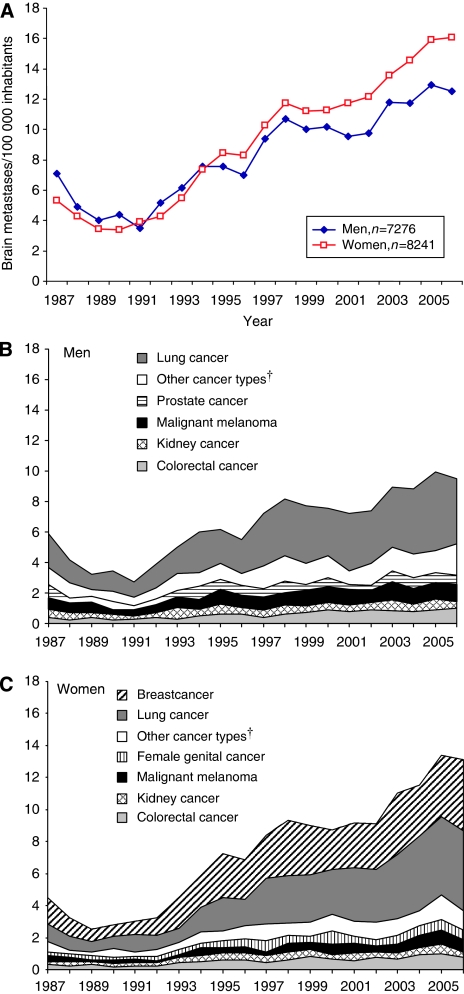
(**A**) Brain metastases admissions in Sweden between 1987 and 2006: age-standardized^*^ rate of patients admitted with BM per 100 000 persons and year. (**B**) Brain metastases admissions in Sweden between 1987 and 2006: age-standardized^*^ rate of male patients admitted with BM per 100 000 persons and year. (**C**) Brain metastases admissions in Sweden between 1987 and 2006: age-standardized^*^ rate of female patients admitted with BM per 100 000 persons and year. ^*^The Swedish population in the year 1995 was used as the standard. ^†^Included mainly cancer in the upper gastrointestinal tract, bladder and ear–nose–throat area.

The incidence of patients hospitalised with BM in Sweden doubled from 7 per 100 000 persons and year in 1987 to 14 per 100 000 persons and year in 2006 (Figure 1). The cohort included more women than men, and about one-third (33%) of the patients were younger than 60 years at first hospitalisation (Table 1). The median age at first admission was 67 years among men and 64 years among women. Half of the patients (50%) were admitted with BM as the primary or main diagnosis, and the other half were admitted with BM secondary to other cancer diagnoses (43%) or to non-cancer disorders/complications (7%).

Among individuals with one primary solid cancer, the most common cancer types in men were lung cancer (44%), malignant melanoma (12%), colorectal cancer (9%) and prostate cancer (9%). In women, lung cancer and breast cancer were equally common (approximately 33%), followed by colorectal cancer (7%) and malignant melanomas (6%). In patients with a history of multiple cancer types, lung cancer was the most common type in both men and women (39 and 41%, respectively, Table 1).

The median time between diagnosis of the primary cancer and first admission with BM varied from 2.6 months in patients with lung cancer to more than 3 years in patients with malignant melanoma or breast cancer, and was almost twice as long among women compared with men (Table 2). Excluding gender-specific malignancies, time to admission was still longer in women compared with men with lung cancer or malignant melanoma, whereas there was no difference by gender among patients with colorectal, kidney or other cancer types when taking age and calendar period differences into account (data not shown). Overall, the time to first admission increased with age, more so in women than in men (men ⩾80 years of age HR 0.81, women ⩾80 years of age HR 0.69, compared with individuals younger than 50 years, Table 2). The time to hospitalisation also increased over successive calendar periods among men (HR 0.85 for the period 2002 to 2006 compared with 1987 to 1991, Table 2), but remained unchanged among women.

Median survival from admission with BM was short, 2.7 months, and did not vary by the number of recorded primary cancers (Table 3). By cancer type, the survival time ranged from 2.5 months among patients with lung cancer to 4.7 months among those with kidney cancer. Similarly, the proportion of patients still alive 1 year after first admission was 13% overall, ranging from 10% in lung cancer patients to 21% in kidney cancer patients. For the vast majority of patients who died during follow-up, the primary cancer type was registered as the main cause of death. High age at admission for BM was a strong predictor of death among both men and women. There was no improvement in the median survival time or the 1-year survival proportion over successive calendar periods. On the contrary, in the adjusted analysis, there was a small increase in risk of death between 2002 and 2006 compared with between 1987 and 1991 in both sexes.

## Discussion

In summary, we observed that the age-adjusted incidence of patients admitted to hospital with BM doubled during the period 1987 to 2006 in Sweden. The primary malignancies behind this increase were mainly lung cancer in both sexes and breast cancer in women. The median time from primary cancer diagnosis to BM admission varied greatly, from 3 months in patients with lung cancer to 42 months in breast cancer patients. However, overall, the time to admission remained largely unchanged over the period of study. In contrast, the survival time from first admission was short, with a median of 3 months, varied little by cancer type, and decreased slightly over calendar time.

In a study from the Netherlands, including 1700 lung or breast cancer patients with BM diagnosed from 1986 to 1998 (Schouten *et al*, 2002), no similar increase in the incidence of BM was noted. The discrepancy between the studies could be attributed not only to different definitions of the population at risk when computing incidence estimates, but also to a shorter time period of observation in the Dutch study. Indeed, we noted an increase in the incidence of BM patients with lung or breast cancer from the middle of the 1990s and onwards, whereas during the earliest period, 1987 to 1990, the incidence decreased.

The annual age-adjusted incidence rate of all primary malignancies in Sweden increased from 490 to 530 per 100 000 individuals during the period 1987 to 2006 (Cancer Incidence in Sweden, 2007). For many cancer types, there has been a parallel improvement in prognosis (Lin and Winer, 2007; Vora and Reckamp, 2008), likely in part because of the earlier detection of indolent tumours (Cancer Incidence in Sweden, 2007), but also because of the introduction of more effective treatment. The prevalence of cancer (ever diagnosed) increased from 3236 per 100 000 in 1997 to 4130 in 2006 in Sweden, in an increasingly older population (Cancer Incidence in Sweden, 1999, 2007). This has plausibly resulted in a higher prevalence of patients at risk of developing metastatic disease.

The incidence rates of lung cancer in many Western countries, including Sweden, have stabilized and even decreased in recent years among men, whereas an increasing trend is still evident among women (Cancer Incidence in Sweden, 2007; Toh, 2009). Brain metastases are a common event in lung cancer, observed in 10–20% of patients screened at diagnosis of a non-small-cell lung cancer (Gavrilovic and Posner, 2005), and even more frequently in small-cell lung cancer patients (Castrucci and Knisely, 2008). With time from lung cancer diagnosis, the proportion of patients developing BM increases rapidly (Mujoomdar *et al*, 2007; Slotman *et al*, 2007). A reported improvement in the short-term survival (Slotman *et al*, 2007; Vora and Reckamp, 2008) may therefore, together with a presumed inefficiency of systemic therapy beyond the blood–brain barrier (Slotman *et al*, 2007), constitute important prerequisites for the increased numbers of lung cancer patients admitted with BM of both sexes in our study. As routine brain scans are seldom performed in asymptomatic patients in Sweden, and prophylactic cranial irradiation is still uncommon, the impact of these measures is limited.

Breast cancer incidence has increased slowly over the past 20 years in Swedish women (Cancer Incidence in Sweden, 2007), partly because of earlier detection through mammography screening, but also because of the lower parity and increasing age of mothers at first birth as well as the widespread use of hormonal replacement therapy among Swedish women (Cancer Incidence in Sweden, 2007). These changes, as well as a general improvement in the prognosis of breast cancer (Cancer Incidence in Sweden, 1999; 2007; Lin and Winer, 2007), provide a basis for the substantial increase observed in breast cancer patients hospitalised with BM. The reported incidence of BM among women diagnosed with breast cancer ranges from 5 to 20% (Schouten *et al*, 2002; Barnholtz-Sloan *et al*, 2004). Characteristics repeatedly associated with an increased BM risk include young age, oestrogen receptor-negative tumours and advanced stage at primary diagnosis (Palmieri *et al*, 2006; Lin and Winer, 2007), whereas the potential predictive value of HER-2 (expressed in 20–30% of breast tumours) is more uncertain (Palmieri *et al*, 2006). The introduction of adjuvant anti-HER-2 antibody treatment, trastuzumab, with low presumed efficiency in the CNS, may have altered the natural history of patients with HER-2-positive breast cancer and unmasked CNS as a potential sanctuary site (Lin and Winer, 2007). This ‘HER-2 paradigm’ may also be relevant for other breast cancer patients in whom successful control of the disease outside of the CNS is achieved with adjuvant or palliative chemo- and radiotherapy (Lin and Winer, 2007).

In spite of a steady increase in incidence of malignant melanomas in Sweden during the investigated period (Cancer Incidence in Sweden, 2007), we did not observe any corresponding increase in melanoma patients admitted with BM. As the increase in primary melanoma incidence may be largely confined to early-stage tumours with very low, if any, metastatic potential (Rees, 2008), the pool of prevalent patients truly at risk of metastatic disease may have increased much less. Alternatively, we may have failed to detect a true modest increase as our study was limited to hospitalised patients. Similarly, the lack of clear time trends for BM patients with colorectal, prostate, kidney or female genital cancer should be interpreted with caution. Prostate cancer was the fourth most common primary malignancy among men admitted with BM in our study. However, we cannot exclude some degree of misclassification of skull or skull base metastases as BM because of the similarity of symptoms. Patients with BM attributed to prostate cancer have not been included in previous studies of BM patients, which limits the possibilities of comparison (Schouten *et al*, 2002; Barnholtz-Sloan *et al*, 2004).

Survival following first admission with BM was on average median shorter than 3 months, and the 1-year survival proportion was only 13%. The highest 1-year survival proportions, around 20%, were observed for patients with kidney, breast or prostate cancer, which is in line with previous reports for breast cancer (Palmieri *et al*, 2006; Altundag *et al*, 2007), whereas studies for prostate cancer are scarce. In a US study of patients with BM from renal cell carcinoma (Shuch *et al*, 2008), 1-year survival was, however, 48%. The discrepancy may be explained by the use of time from hospital admission to death in our study rather than from diagnosis, but also perhaps by differences in CNS-screening routines between the United States and Sweden.

Our study covered two decades and was based on comprehensive reporting of all hospital admissions and primary malignancies, and on a virtually complete follow-up. To our knowledge, no national studies of patients with BM encompassing all primary cancer types have been published previously. Our data give important information about an expanding group of patients with a significant need for supportive care and thus generating high health-care costs (Taillibert and Delattre, 2005). The study was, however, limited to patients hospitalised with BM. Although it is feasible to believe that the majority of patients diagnosed with BM are hospitalised at some point during the disease course, our cohort most likely represents an underestimation of the number of BM patients in the population. Also, temporal changes in the society and in health-care routines need to be taken into account when interpreting the observed time trend. The increasing use of advanced neuroimaging techniques may have inflated the number of identified BM patients. However, more conservative hospital admission practices attributed to a general reduction of inpatient beds could have had a counteracting effect. The introduction of new treatment options for certain patient groups may have affected admission rates differentially. However, any temporal changes in admission or registration routines driven by socioeconomic or political factors should have affected all cancer patients alike and are less likely to explain the observed differences in time trends by cancer type.

Another concern is that we did not have confirmatory information about the diagnostic workup or biopsy results linking the primary cancer to the brain lesions. For patients registered with multiple tumours, the primary cancer diagnosed closest in time before the date of first hospitalisation for BM was regarded as the cancer causing BM, without validation through medical reports. However, the distribution of cancer types among those patients was similar to that of patients with only one primary cancer record. An additional 12% of patients admitted with BM were not recorded with any primary malignancy in the Swedish Cancer Register. This group represents a mixture of patients with unknown, or known but unreported, primary malignancies. A degree of underreporting higher than average to the NCR has been described for patients with lung and digestive cancer, in validations against the Cause-of-Death Registry (Cancer Incidence in Sweden, 2007).

To conclude, we found a doubled incidence of patients admitted with BM during the 20-year long study period. The largest increases were observed in patients with lung cancer and breast cancer. Considering the recent improvements in the prognosis of patients with lung cancer and breast cancer, the increasing efficiency in controlling disease spread outside of the CNS, and the short survival of advanced cancer patients with BM, therapeutic interventions aiming at reducing the risk of BM are essential.

## Figures and Tables

**Table 1:** Brain metastases (BM) admissions in Sweden between 1987 and 2006: characteristics of men and women admitted with BM and type of primary cancer

	**Men**				**Women**			
		**Primary cancer in the Cancer Registry**				**Primary cancer in the Cancer Registry**		
**Characteristic**	**Total**	**One cancer**	**More than one cancer**	**No primary cancer^a^**	**Total**	**One cancer**	**More than one cancer**	**No primary cancer^a^**
*Total*	7276	5672	687	917	8241	6635	737	869
Row % by sex		78.0	9.4	12.6		80.5	8.9	10.5
								
*Age at first admission (years)*								
Median	67	66	72	70	64	62	68	70
Q-range	59–74	58–73	66–78	61–77	55–72	54–71	60–75	58–79
								
	**Number**	**Row %**			**Number**	**Row %**		
<50	576	89.1	1.9	9.0	1182	90.9	2.3	6.9
50–59	1339	85.1	4.3	10.6	2000	85.7	6.6	7.8
60–69	2318	81.1	8.2	10.7	2325	81.0	10.5	8.5
70–79	2254	72.2	13.3	14.5	1961	75.2	12.3	12.5
80+	789	65.0	16.1	18.9	773	63.4	12.0	24.6
								
*Calendar period*								
1987–1991	901	82.8	6.4	10.8	802	80.0	7.5	12.5
1992–1996	1417	81.1	7.8	11.2	1491	82.1	8.5	9.5
1997–2001	2196	76.1	9.2	14.7	2547	79.1	9.1	11.8
2002–2006	2762	76.2	11.5	12.3	3401	81.0	9.4	9.6
								
		**Column %**				**Column %**		
*Type of primary cancer* b								
Lung cancer		44.1	39.4			33.5	41.0	
Breast cancer		—	—			32.6	15.1	
Colorectal cancer		9.0	10.5			7.4	7.2	
Malignant melanoma		12.3	6.4			5.8	5.8	
Female genital cancer		—	—			5.6	8.8	
Kidney cancer		7.8	7.3			4.7	5.4	
Prostate cancer		8.6	13.1			—	—	
Other solid cancers^c^		18.2	23.3			10.5	16.7	

a Patients with no solid cancer diagnosis in the Swedish Cancer Register.

b For patients with more than one primary solid cancer reported to the Swedish Cancer Register, the diagnosis closest in time to the date of admission with BM was chosen-.

c Included mainly cancer in the upper gastrointestinal tract, bladder and ear–nose–throat area.

**Table 2:** Brain metastases (BM) admissions in Sweden between 1987 and 2006: time from primary solid cancer diagnosis to first admission with BM^a^

			**Adjusted time^b^ to admission with BM**	
		**Time to admission with BM (months)**	**Men**	**Women**
**Characteristic**	**n**	**Median (Q-range)**	**HR (95% CI)^b^**	**HR (95% CI)^b^**
*Type of primary cancer*	12 307	12.2 (1.2–37.0)		
Lung cancer	4724	2.6 (0.0–10.2)	3.15 (2.86–3.48)	2.92 (2.64–3.23)
Breast cancer	2163	41.7 (20.4–78.0)	—	0.52 (0.47–0.58)
Colorectal cancer	1001	25.2 (10.5–45.5)	1.00 (reference)	1.00 (reference)
Malignant melanoma	1087	38.6 (20.0–69.9)	0.74 (0.66–0.83)	0.48 (0.42–0.55)
Female genital cancer	370	25.8 (11.5–56.6)	—	0.63 (0.55–0.73)
Kidney cancer	749	17.0 (3.1–47.8)	1.02 (0.90–1.16)	0.90 (0.78–1.04)
Prostate cancer	485	27.2 (10.2–61.4)	0.90 (0.80–1.02)	—
Other solid cancers^c^	1728	5.2 (0.0–21.1)	1.50 (1.35–1.67)	1.44 (1.28–1.62)
				
*Sex*				
Men				
Women	5672	8.8 (0.5–28.3)		
	6635	16.3 (2.6–45.5)		
				
*Age*^d^ *(years)*				
<50	1587	18.2 (4.7–43.2)	1.00 (reference)	1.00 (reference)
50–59	2852	11.5 (1.3–34.3)	1.02 (0.92–1.14)	0.83 (0.77–0.89)
60–69	3762	10.4 (0.9–31.5)	0.99 (0.89–1.09)	0.74 (0.68–0.80)
70–79	3103	11.3 (0.9–38.5)	0.92 (0.83–1.02)	0.67 (0.62–0.73)
80+	1003	17.5 (1.5–51.7)	0.81 (0.71–0.92)	0.69 (0.62–0.77)
				
*Calendar period*				
1987–1991	1388	11.8 (0.2–35.0)	1.00 (reference)	1.00 (reference)
1992–1996	2373	13.8 (1.1–40.2)	0.91 (0.83–1.00)	1.03 (0.94–1.14)
1997–2001	3687	11.5 (1.2–35.3)	0.91 (0.84–1.00)	1.04 (0.95–1.13)
2002–2006	4859	12.1 (1.7–37.1)	0.85 (0.79–0.93)	1.02 (0.93–1.11)

a The analyses were restricted to BM patients with only one primary solid cancer recorded (12 307 out of 15 517, 79%).

b Time from the primary solid cancer diagnosis to admission with BM computed as hazard ratio (HR) and 95% confidence interval (CI) in a multivariate Cox regression model including adjustment for age, sex and calendar period, except in analyses of main effects of each covariate.

c Included mainly cancer in the upper gastrointestinal tract, bladder and ear–nose–throat area.

d Age at first admission to hospital with BM.

**Table 3:** Brain metastases (BM) admissions in Sweden between 1987 and 2006: survival from first admission with BM

		**Proportion deceased**	**Survival (months)**	**One-year survival proportion**		**Relative risk^a^ of death following admission with BM**	
	**Number**	**%**	**Median (Q-range)**	**%**	**(95% CI)**	**Men HR (95% CI)^a^**	**Women HR (95% CI)^a^**
Total	15 517	97.5	2.7 (1.2–6.5)	13.0	(12.5–13.6)		
							
*One primary cancer*	12 307	97.8	2.7 (1.2–6.7)	13.3	(12.7–13.9)		
Lung cancer	4724	98.7	2.5 (1.2–5.9)	10.4	(9.5–11.3)	1.07 (0.97–1.18)	1.03 (0.93–1.14)
Breast cancer	2163	96.9	3.2 (1.2–9.1)	19.0	(17.4–20.7)	—	0.92 (0.83–1.01)
Colorectal cancer	1001	99.0	2.6 (1.4–5.1)	6.7	(5.3–8.4)	1.00 (reference)	1.00 (reference)
Malignant melanoma	1087	97.9	2.7 (1.1–5.9)	9.8	(8.2–11.7)	1.08 (0.96–1.22)	1.03 (0.90–1.17)
Female genital cancer	370	97.0	2.9 (1.3–7.8)	18.4	(14.6–22.4)	—	0.87 (0.76–1.00)
Kidney cancer	749	96.4	4.7 (2–10.3)	21.1	(18.3–24.1)	0.69 (0.60–0.78)	0.67 (0.58–0.78)
Prostate cancer	485	97.7	2.9 (1.2–8.0)	19.4	(16.0–23.0)	0.72 (0.63–0.81)	—
Other solid cancers^b^	1728	96.4	2.5 (1.1–6.3)	13.7	(12.1–15.3)	0.98 (0.88–1.09)	0.94 (0.83–1.05)
							
More than one cancer	1424	98.2	2.4 (1.1–5.6)	9.7	(8.2–11.3)	1.01 (0.90–1.13)	0.91 (0.81–1.02)
No primary cancer^c^	1786	94.9	2.6 (1.3–6.3)	14.2	(12.6–15.9)	0.85 (0.76–0.95)	0.79 (0.71–0.89)
							
*Sex*							
Men	7276	97.8	2.5 (1.2–6.0)	11.4	(10.7–12.1)		
Women	8241	97.2	2.9 (1.3–7.2)	14.4	(13.7–15.3)		
							
*Age* d							
<50	1758	93.8	4.5 (1.6–11.4)	23.6	(21.6–2.6)	1.00 (reference)	1.00 (reference)
50–59	3339	97.0	3.5 (1.4–8.8)	18.0	(16.7–19.3)	1.24 (1.12–1.37)	1.18 (1.09–1.27)
60–69	4643	97.5	2.9 (1.3–6.8)	12.9	(11.9–13.9)	1.44 (1.31–1.58)	1.36 (1.26–1.46)
70–79	4215	98.9	2.2 (1.1–4.6)	8.1	(7.3–8.9)	1.89 (1.71–2.08)	1.70 (1.57–1.83)
80+	1562	98.6	1.8 (0.9–3.2)	4.6	(3.6–5.7)	2.42 (2.16–2.72)	2.17 (1.97–2.39)
							
*Calendar period*							
1987–1991	1703	98.2	3.6 (1.6–8.3)	17.5	(15.7–19.3)	1.00 (reference)	1.00 (reference)
1992–1996	2908	98.9	3.0 (1.3–7.6)	14.8	(13.5–15.1)	1.05 (0.97–1.14)	1.11 (1.02–1.21)
1997–2001	4743	98.3	2.5 (1.2–6.1)	11.8	(10.9–12.7)	1.17 (1.08–1.27)	1.19 (1.10–1.29)
2002–2006	6163	96.0	2.5 (1.1–5.9)	12.0	(11.2–12.8)	1.18 (1.09–1.27)	1.18 (1.09–1.28)

a Relative risk of death following admission with BM computed as hazard ratio (HR) and 95% confidence interval (CI) in a multivariable Cox regression model compared with a reference.

b Included mainly cancer in the upper gastrointestinal tract, bladder and ear–nose–throat area.

c Patients with BM in the Patient register but with no solid cancer in the Swedish Cancer Register.

d Age at first admission with BM.
